# Statin utilization and its predictors for the primary prevention of cardiovascular disease among type 2 diabetic patients in a resource-limited setting

**DOI:** 10.3389/fendo.2025.1472300

**Published:** 2025-07-04

**Authors:** Feyisa Assefa Dhaba, Wubshet Abraham Alemu, Firaol Lamessa Kitila, Koricho Simie Tolla, Astarekew Alene, Zeru Seyoum Wendimagegn, Kedir Ali Getahun, Hassen Ahmed Yesuf, Surafel Mekasha Woldeyes

**Affiliations:** ^1^ Department of Internal Medicine, School of Medicine, College of Medicine and Health Sciences, Assela University, Assella, Ethiopia; ^2^ Department of Internal Medicine, School of Medicine, College of Medicine and Health Sciences, Woldia University, Woldia, Ethiopia; ^3^ Department of Gynaecology and Obstetrics, School of Medicine, College of Medicine and Health Sciences, Woldia University, Woldia, Ethiopia; ^4^ Department of Midwifery, College of Medicine and Health Sciences, Woldia University, Woldia, Ethiopia; ^5^ Department of Biomedical Science, School of Medicine, College of Medicine and Health Sciences, Woldia University, Woldia, Ethiopia

**Keywords:** diabetes mellitus, statin, primary prevention, cardiovascular disease, Ethiopia

## Abstract

**Background:**

Cardiovascular disease (CVD) is a major cause of mortality in patients with type 2 diabetes mellitus (T2DM). Statins are essential for the primary prevention of CVD in this high-risk group. Despite guideline recommendations, statin use remains suboptimal in clinical practice. Assessing statin utilization and identifying factors influencing their prescription are vital for enhancing evidence-based care, especially in resource-limited settings. Therefore, this study aimed to evaluate statin use and its predictors for primary CVD prevention among diabetic patients at Arsi Teaching and Referral Hospital in 2023.

**Methods:**

A hospital-based cross-sectional study was carried out among 351 diabetic patients at Arsi Teaching and Referral Hospital (ARTH) between February and September 2022. Participants were selected using a systematic random sampling method. Data were collected through a pretested, interviewer-administered structured questionnaire and a checklist. Trained nurses from the diabetic clinic conducted data collection. The collected data were initially entered into Epi Data version 7.2, then exported to SPSS version 26 (IBM, USA) for analysis. The association between statin use and potential predictors was examined using odds ratios (ORs) with 95% confidence intervals (CIs). Variables with a p-value below 0.05 in the multivariable logistic regression were considered statistically significant.

**Results:**

The prevalence of statin utilization for the primary prevention of CVD among type 2 diabetic patients on follow-up at Asella Teaching Hospital was 189 [(53.8%, 95% CI: 48.6%, 59.0%)]. The most commonly prescribed statin was atorvastatin, followed by simvastatin. The probability of statin use was greater in participants with hypertension [AOR=4.8, 95% CI (2.0-11.5)], dyslipidemia [AOR=10.5, 95% CI (2.0-11.5)], and uncontrolled glycemic control [AOR=8.0,95% CI (3.8-17.7)].

**Conclusions:**

Suboptimal statin utilization (53.8%) for primary CVD prevention in type 2 diabetes was observed, with utilization heavily influenced by comorbidities. Urgent quality improvement initiatives are needed to ensure statin access aligns with guideline recommendations for patients with hypertension, dyslipidemia, and poor glycemic control in the study area.

## Introduction

### Background

Diabetes mellitus (DM) is a chronic and progressive metabolic disorder resulting from inadequate insulin secretion or the body’s ineffective use of insulin (1, 2). According to a report published by the International Diabetes Federation (IDF) in 2021, the global prevalence of diabetes among individuals aged 20 to 79 years is estimated to be approximately 537 million. However, this number is expected to rise significantly, reaching approximately 643 million by 2030 and a staggering 783 million by 2045 (3). Diabetes mellitus significantly increases the risk of cardiovascular disease (2, 4).

Cardiovascular diseases (CVDs) are the leading cause of morbidity and mortality among patients with type 2 diabetes mellitus (T2DM) (5). This increased risk is largely due to higher concentrations of low-density lipoprotein (LDL) particles in diabetic patients. These LDL particles tend to be smaller, denser, and more susceptible to oxidation, which enhances the risk of cardiovascular events (6, 7). Therefore, managing cardiovascular risk factors like dyslipidemia is vital to lowering the risk of heart-related complications in people with diabetes. As a result, several clinical practice guidelines have outlined strategies to prevent these complications. One of the key recommendations is the use of lipid-lowering therapies (LLTs), particularly statins, which play a significant role in reducing cardiovascular disease (CVD) among diabetic patients (5, 8, 9).

Statins are lipid-lowering agents that competitively inhibit HMG-CoA reductase, the rate-limiting enzyme in hepatic cholesterol biosynthesis. This inhibition directly reduces cholesterol synthesis (9). Statins are divided into three intensity levels based on their LDL cholesterol-lowering capacity: high-intensity statins reduce LDL levels by over 50%, moderate-intensity statins lower them by 30% to 50%, and low-intensity statins decrease LDL by less than 30% (10, 11).

The American Diabetes Association (ADA) recommends moderate-intensity statin therapy for all individuals with type 2 diabetes (T2D) aged 40 to 75 years as a measure for primary prevention (12). Similarly, the American College of Cardiology and the American Heart Association (ACC/AHA) advise that patients within this age range who have T2D and an LDL-C level of ≥70 mg/dL should start moderate-intensity statins, without the need to assess their 10-year ASCVD risk (13). These recommendations highlight the crucial role of statins in preventing cardiovascular events in people with T2D. Numerous studies have also supported the effectiveness of statins in lowering cardiovascular events and mortality in diabetes. A population-based study in Korea found that statin use in diabetic patients reduced the risk of myocardial infarction, stroke, and mortality by 28% (14). Despite this, statin use remains low: only 37.7% of diabetic patients in the USA (15) and 33.8% in Hong Kong (16) received prescriptions. In sub-Saharan Africa, data are limited, but studies in Ethiopia show low prescription rates of 36.7% in Jimma (17), and 40% in Bonga (18). Statin utilization for primary CVD prevention is influenced by multifaceted determinants: patient demographics (age, sex, education, diabetes duration), clinical comorbidities (hypertension, dyslipidemia, uncontrolled glycemia), and systemic factors (medication access, provider prescribing patterns, and guideline adherence) (17–20).

As the global prevalence of diabetes continues to rise (21), Different international guidelines strongly recommend the use of statins for the primary prevention of cardiovascular events in individuals with type 2 diabetes. However, their actual implementation in clinical practice remains suboptimal in many healthcare systems. This gap is particularly concerning in low- and middle-income countries, where the diabetes burden is rapidly increasing. In Ethiopia—specifically in our study area—there is limited and inconsistent data regarding statin utilization and its predictors. Therefore, this study aims to assess the extent of statin utilization and identify its key predictors among diabetic patients attending Arsi Teaching and Referral Hospital, a major facility serving a large diabetic population. Assessing statin utilization patterns and their determinants at Arsi Hospital provides critical insights for developing targeted strategies to improve cardiovascular risk prevention among individuals with diabetes. This localized evidence will support healthcare professionals and policymakers in making evidence-based decisions that optimize patient care, strengthen adherence to clinical guidelines, and reduce the cardiovascular burden associated with diabetes. Furthermore, improving statin use can help reduce long-term healthcare costs by preventing expensive hospitalizations and complications. The findings will also serve as a valuable benchmark for future researchers interested in this area of study.

## Materials and methods

### Study design and setting

A hospital-based cross-sectional study was conducted at Arsi Referral and Teaching Hospital, Arsi University, from January 1, 2022, to August 30, 2022. The hospital is located in the Arsi Zone of Oromia, approximately 175 km from the Ethiopian capital, Addis Ababa. At the outpatient level, the diabetic clinic provides treatment and checks by trained nurses, medical interns, residents, and specialists (22).

### Population

The study population included all adult type two diabetes mellitus patients who underwent diabetic follow-up at Arsi Teaching and Referral Hospital, while adult type two diabetes mellitus patients who underwent diabetic follow-up at Arsi Teaching and Referral Hospital during the study period were considered the study population.

### Sample size and sampling technique

The sample size was calculated based on the single population proportion formula using a 36.67% incidence of statin utilization among diabetic patients from a study conducted in Jimma (17), a 5% margin of error, a 95% confidence level, and a 10% non-response rate.


$$\text{n}\;=({\text{z}}_{\text{α}/2)2^{\text{p}}}\left(1-\text{p}\right)/{\text{d}}^{2}$$

$$\text{n}\;=\left(1.96\right){\;}^{2}\text{x}\left(0.37\right)\text{x}\left(1-0.37\right)/0.05{\;}^{2}=358$$

With adjustment for 5% nonresponse (n=358 + 18), the final sample size was 376

where n is the sample size

z = value of the standard normal distribution corresponding to 95% CI

p = expected proportion in the population

q = 1 - p = 1 - 0.37 = 0.63

d = desired degree of precision 0.05

Finally, a systematic random sampling technique was employed to recruit the required sample size. The value of K was calculated by dividing the estimated total number of diabetic patients who visited the hospital’s diabetic follow-up clinic during the study period (approximately 4800 patients for eight months) by the total number of required samples (376). Then, samples were selected every thirteen (13) intervals based on their order of clinic visits, while the first study subject was chosen by the lottery method (which was the second case).

### Eligibility criteria

All adult type two diabetes mellitus patients who underwent diabetic follow-up at Arsi Teaching and Referral Hospital were included, while diabetic patients who were less than 40 years old, patients taking statins for secondary/tertiary prevention of CVD, and pregnant patients were excluded from the study.

Study variables: The dependent variable was statin utilization, while sociodemographic factors, lifestyle parameters, comorbidities, clinical profiles, anthropometric measurements, and diabetes-related complications were the independent variables.

### Operational definition

Statin utilization was assessed by reviewing documented prescriptions issued throughout the patients’ clinical follow-up period. High-intensity statin therapy was classified as atorvastatin at doses of 40–80 mg or rosuvastatin at 20–40 mg, while moderate-intensity therapy included atorvastatin 10–20 mg or rosuvastatin 5–10 mg (23).

Uncontrolled blood glucose level: Fasting blood sugar level greater than 130 mg/dL (17).

Dyslipidemia: It was defined by any of the following lipid abnormalities: total cholesterol levels of ≥200 mg/dL, LDL-C ≥ 100 mg/dL, triglycerides ≥150 mg/dL, HDL-C ≤ 40 mg/dL in males, or HDL-C ≤ 50 mg/dL in females (24, 25).

### Data collection procedures

A structured interviewer-administered questionnaire was developed by reviewing different literature in English, translated into Afan Oromo (the local language), and then retranslated back into English to check its consistency. Finally, the data were collected using the Afan Oromo version of the questionnaire by three trained nurse professionals who work at the diabetic clinic of the hospital. A review of medical records was performed to collect clinical data using a prepared checklist.

### Anthropometric measurements

Physical measurements: Height and weight were measured using an inelastic measuring tape and weight scale, respectively. Repeated blood pressure measurements were performed at the hospital by trained nurse professionals using standardized operating procedures after the participants rested for approximately five minutes. The height (to the nearest 0.1 cm), weight (to the nearest 0.1 kg), hip and waist circumferences (to the nearest 0.1 cm), and BP (to the nearest 0.5 mmHg) of the patients were measured.

### Data quality assurance

To ensure the quality of the collected data, the questionnaire was translated from English to Afan Oromo (a local language) and then retranslated back to English to maintain consistency. The questionnaire was also pretested with 5% of the diabetic patients at Adama Teaching Hospital. Furthermore, one day training was given to the data collectors and supervisors. Furthermore, the primary investigator visited the data collectors once a day to ensure that the data were collected correctly.

### Data management and statistical analysis

The collected data were checked for completeness, coded, entered into Epi Data 7.2 software, cleaned, and exported to SPSS version 26 (IBM, USA) for statistical analysis. The Kolmogorov–Smirnov test and box plots were used to examine the data distribution and detect outliers. The results of the descriptive analyses are presented as frequencies or means ± SDs, depending on the nature of the data. Finally, the results are presented in the form of tables, figures, and narratives. The Hosmer–Lemeshow test and the multicollinearity test were used to measure model fitness. A chi-square test was used to analyze the associations between categorical variables. The strength of the association between different independent factors and statin utilization was assessed using odds ratios (ORs) and 95% confidence intervals (CIs) from logistic regression. Variables with p-values< 0.25 were considered candidate variables for multivariable logistic regression. Finally, variables with a *P* value less than 0.05 in multivariate logistic regression were considered to be significantly different.

### Ethical considerations

This study was reviewed and approved by the Institutional Review Board (IRB) of Arsi University on behalf of the College of Health Science Research Ethical Committee. A signed permission letter was obtained from the hospital medical director. Finally, participants were informed about the study’s aims, objectives, and potential outcomes. Written informed consent was obtained before the data collection started. All the information obtained in the study was kept confidential, and no records identifying patients were recorded. There was no payment for participating in the study.

## Results

### Sociodemographic, clinical, and lifestyle characteristics

This study included 351 randomly selected study participants, for a response rate of 93.4%, of whom more than half (213, 60.7%) were men. The majority of the study participants (207, 59%) were aged 40–60 years, while 323 (92%) of the participants were married. In terms of respondents’ residency, 212 (60.4%) of the participants lived in urban areas. The occupations of the study participants were also reported. Approximately one-quarter (104, 29.6%) of the participants were farmers, while 78 (22.2%) were housewives. Furthermore, 327 (93.2%) of the study participants had government insurance (**Table 1**).

**Table 1:** Socio-demographic characteristics of type 2 DM patients on follow-up at ARTH in Asella, Oromia, Ethiopia, 2022 (n=351).

Variables	Category	Frequency (%)
Age (years)	40-60	207 (59.0)
	61-75	144 (41.0
Sex	Females	138 (39.3)
	Males	213 (60.7)
Residence	Rural	139 (39.6)
	Urban	212 (60.4)
Marital Status	Married	323(92.0)
	Single	3 (0.9)
	Divorced	16 (4.6)
	Widowed	9 (2.6)
Education level	Never attended school	121 (34.5)
	Primary School	107 (30.5)
	Secondary School	49 (14.0)
	College/University	74 (21.1)
Occupation	Farmers	104 (29.6)
	Housewives	78 (22.2)
	Private jobs	97(27.6)
	Retired	33 (9.4)
	Government employee	39 (11.1)
Government insurance	Yes	327 (93.2)
	No	24 (6.8)

### Clinical characteristics of the study participants

The clinical characteristics of the study participants were also reported. More than half of the 204 (58.1%) participants had more than five years of diabetes history. The most prevalently reported comorbidity was dyslipidemia, which was present in 193 (63.5%) participants, followed by hypertension in 190 (54.1%) participants and chronic kidney disease (CKD) in 38 (11.8%) participants. Similarly, more than half of the study participants, 219 (62.4%) and 205 (58.4%), had blood pressure below 140/90 mmHg and a normal BMI (18.5–25 kg/m²), respectively. Moreover, nearly two-thirds of the 250 participants (71.2%) had a normal waist-to-hip ratio (WHR). Screening for diabetic complications was also reported. Nearly two-thirds of the patients (233, 66.4%) were screened for diabetic retinopathy, and 52 (22.3%) had complications. However, diabetic nephropathy was detected in only 2 (0.6%) patients. Peripheral neuropathy was also found in 66 (18.8%) of the study participants (**Table 2**).

**Table 2:** Clinical characteristics of selected type 2 DM patients on follow-up at ARTH Asella, Ethiopia, 2022 (n=351).

Variables	Category	Frequency	Percent
Diabetes duration	<5 years	147	41.9
	≥5 years	204	58.1
Blood Pressure (mmHg)	<140/<90	219	62.4
	≥140/90	132	37.6
WHR	>0.9 (M) or >0.85(F)	101	28.8
	≤0.9 (M) or ≤0.85 (F)	250	71.2
BMI (kg/m²)	<18.5	9	2.6
	18.5-25	205	58.4
	25-30	87	24.8
	>30	50	14.2
Hypertension	Yes	190	54.1
	No	161	45.9
Dyslipidemia	Yes	193	63.5
	No	111	36.5
CKD	Yes	38	11
	No	307	89.0
Diabetic retinopathy	Yes	52	22.3
	No	181	77.7
Peripheral neuropathy	Yes	66	18.8
	No	285	81.2
Diabetic nephropathy	Yes	2	0.6
	Never screened	349	99.4

WHR, Weight-to-hip ratio; BMI, Body mass index; CKD, Chronic kidney disease.

### Behavioral characteristics of the study participants

The behavioral characteristics of the study participants were also reported; 19 (5.4%) and 42 (31.7%) had past histories of smoking and alcohol consumption, respectively. The majority of participants (212, 60.4%) had never performed physical exercise (**Table 3**).

**Table 3:** Behavioral characteristics of selected type 2 DM patients at follow-up at ARTH Asella, Ethiopia, 2022 (n=351).

Variables	Category	Frequency	Percent
Drinking alcohol	Never	307	87.5
	Sometimes	42	12.0
	Regularly	2	0.6
Cigarette smoking	Never	330	94.0
	Current smoker	2	0.6
	Former smoker	19	5.4
Physical exercise	Regularly	26	7.4
	Sometimes	113	32.2
	Never	212	60.4

### Clinical parameters of the study participants

Regarding the clinical parameters, the lipid profile was determined in 304 (86.6%) of the participants, where the most common lipid abnormality was a low level of high-density lipoprotein (HDL) in 134 (44.1%), followed by high triglycerides in 121 (39%) and high LDL in 104 (34.2%). In addition, high total cholesterol was reported in one-third of the 111 (36.5%) patients. Based on fasting blood glucose levels, the majority, 199 (56.7%), of the patients had an uncontrolled glucose level (≥130 mg/dl), while one-third, 114 (32.5%), of the patients had a HgA1C concentration >8%, 70 (19.9%) of whom had an uncontrolled glucose level (**Table 4**).

**Table 4:** Clinical parameters of type 2 DM patients at follow-up at ARTH, Asella, Ethiopia, 2022 (N=351).

Variables	Category	Frequency	Percent
Lipid profile	Yes	304	86.6
	No	47	13.4
LDL	≥130	104	34.2
	<130	200	65.8
HDL	≤40(M) ≤ 50(F)	134	44.1
	>41 (M) >51(F)	170	55.9
TC	≥200	111	36.5
	<200	193	63.5
TG	≥150	121	39.8
	<150	183	60.2
Average FBS over three consecutive visits	<130	152	43.3
	≥130	199	56.7
HgA1C (%)	<8%	44	12.5
	≥8%	70	19.9
	Total	114	32.5
RFT (eGFR)	>60 ml/min/1.73m2	307	89.0
	45–60 ml/min/1.73 m²	26	7.5
	30–45 ml/min/1.73 m²	11	3.2
	15–30 ml/min/1.73 m²	1	0.3

All lipid profile parameters and fasting blood glucose level were measured in a unit of mg/dl, and eGFR was measured by mL/min/1.73 m². LDL, Low-Density Lipoprotein; HDL, High-Density Lipoprotein; FBS (in mg/dL), TC, Total Cholesterol; TG, Triglycerides; Fasting Blood Sugar; HbA1C, Hemoglobin A1C; RFT, Renal Function Test; and eGFR, Estimated Glomerular Filtration Rate.

**Figure 1:**
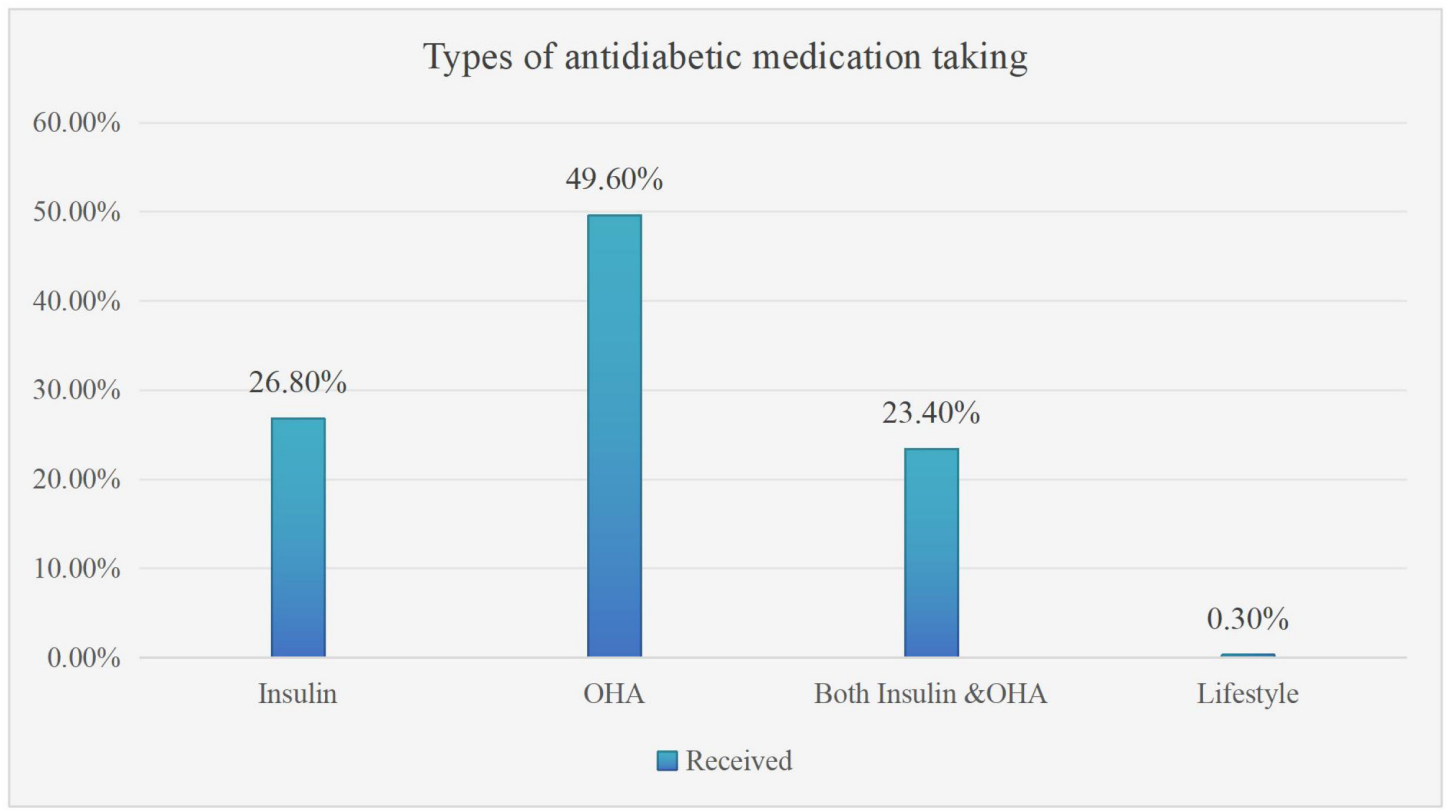
Medication for diabetes in selected type 2 DM patients on follow-up at ARTH Asella, Ethiopia, 2022 (n=351). OHA stands for oral hypoglycemic agents (metformin, glibenclamide, and others), and lifestyle modification includes regular exercise and diet modification.

Regarding the types of hypoglycemic agents, almost half of the diabetic patients, 174 (49.6%), were treated with oral hypoglycemic agents, while 94 (26.7%) and 82 (23.4%) were treated with insulin and both insulin and oral hypoglycemic agents, respectively (**Figure 1**).

### Statin utilization for primary prevention of cardiovascular diseases

In this study, statins were prescribed to more than half 189 (53.8%) of the study participants; the most commonly prescribed statin was atorvastatin 40 mg per day in 164 (86.3%) participants, followed by atorvastatin 20 mg in 12% of participants (**Table 5**).

**Table 5:** Statin prescriptions for the primary prevention of cardiovascular diseases for type 2 DM patients on follow-up at ARTH, Asella, Ethiopia, 2022 (N=351).

Variables	Category	Frequency	Percent
Statin prescription	No	162	46.2
	Yes	189	53.8
Type and dose of statin	Atorvastatin 20 mg	24	12.6
	Atorvastatin 40 mg	163	86.3
	Simvastatin 20 mg	2	1.1

**Figure 2:**
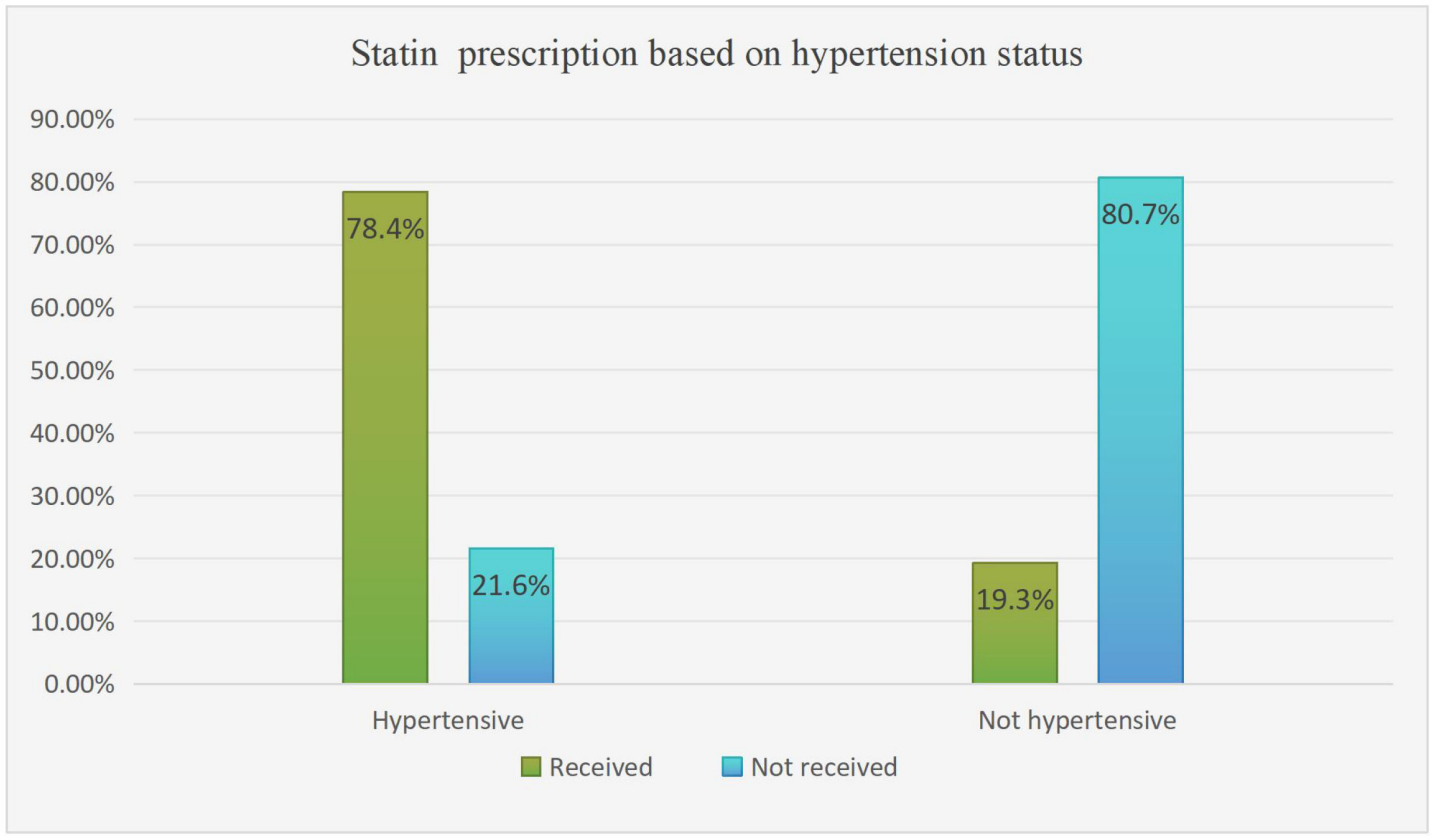
Statin utilization pattern based on hypertension status among type 2 DM patients on follow-up at ARTH, Asella, Ethiopia, 2022 (N=351).

**Figure 3:**
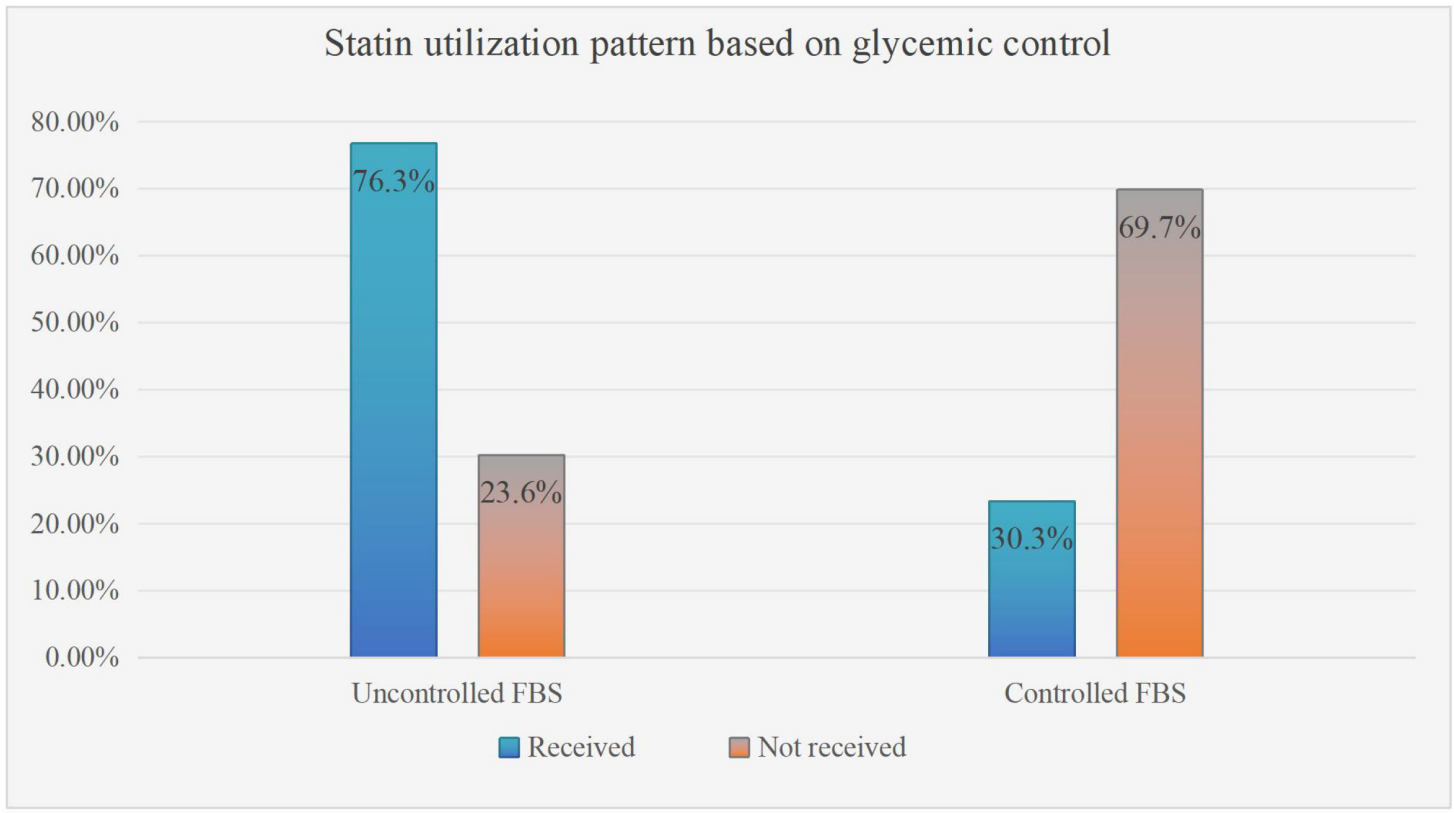
Statin utilization pattern based on glycemic control of type 2 DM patients on follow-up at ARTH, Asella, Ethiopia. September 2022 (N=351). Note: Uncontrolled FBS stands for fasting blood glucose (FBS) level >126 mg/dl.

Among hypertensive study participants, more than two-thirds 149 (78.4%) were prescribed a statin. However, non-hypertensive patients made up only 31 (19.3%) of the patients (**Figure 2**). Based on glycemic control status, 152 (76.3%) patients with uncontrolled glycemic control were prescribed statins, whereas 46 (30.3%) of the participants had controlled FBS (**Figure 3**).

### Predictors of statin utilization for the primary prevention of cardiovascular events among diabetic patients

According to bivariate logistic regression, age, duration of diabetes (years), waist-to-hip ratio, body mass index (BMI), government insurance status, hypertension status, dyslipidemia status, high FBS, high LDL, low HDL, high total cholesterol, hypertriglyceridemia status, and type of treatment for diabetes were fitted into a multivariable binary logistic regression model for further analysis. The multivariable analysis results showed that hypertension, dyslipidemia, and uncontrolled hyperglycemia remained significantly associated with statin utilization.

According to this study, hypertensive participants were found to have an approximately five-fold greater likelihood of being on statins [AOR=4.837, 95% CI (1.936-11.587)] than those who were not hypertensive. Similarly, the odds of using statins were almost ten times greater among participants with abnormal lipid profiles (dyslipidemia) [AOR=10.5, 95% CI (2.185–11.116)] as compared to those with a normal lipid profile. In addition, patients with uncontrolled blood sugar levels were also eight times more likely to use statins [AOR=8.049, 95% CI (3.87-17.7)] than patients with controlled glycemic levels (**Table 6**).

**Table 6:** Determinants of statin utilization in selected type 2 DM patients on follow-up at ARTH, Asella, Ethiopia, 2022 (N=351).

Variables	Category	Statins utilization				
		Yes	No	COR (95% CI)	AOR (95% CI)	P value
Age (years)	40-60	104	103	1	1	
	60-75	85	59	1.4 (1.1-2.3)	1.1 (0.6-2.4)	0.767
Duration of diabetes (years)	<5 years	60	87	1		
	≥5 years	129	75	2.5 (1.6-3.9)	1.8 (0.86-4.0)	0.128
WHR	>0.9 (M) or >0.85 (F)	86	15	8.2 (4.5-15)	2.1 (0.5-8.1)	0.280
	<0.9 (M) or<0.85 (F)	103	147	1	1	
BMI (kg/m²)	<18.5	4	5	0.1 (0.02-0.4	0.6 (0.4-8.6)	0.687
	18.5-25	68	137	0.05 (0.0-0.1)	0.2 (0.03-1.2)	0.057
	25-30	72	15	0.5 (0.2-1.6)	0.97 (0.2-4.77)	0.971
	>30	45	5	1	1	
Insurance	Yes	182	145	3.0 (1.2-7.5)	1.6 (0.28-9.2)	0.602
	No	7	7	1	1	
Dyslipidemia	Yes	142	51	6.3 (3.8-10.6)	10.5 (2-11.5)	0.001*
	No	34	77	1	1	
Hypertension	Yes	149	41	11 (6.7-18.1)	4.8 (2.0-11.5)	0.000*
	No	40	121	1	1	
Average FBS	≥130	145	49	7.6 (4.7-12.2)	8 (3.8-17.7)	0.000*
	<130	44	113	1	1	
LDL	≥130	72	32	2.1 (1.3-3.4)	0.62 (0.24-1.6)	0.316
	<130	104	96	1	1	
HDL	≤40 (M), ≤ 50 (F)	95	39	2.6 (1.6-4.3)	0.6 (0.2-1.7)	0.390
	>41 (M), >51 (F)	81	89	1	1	
TC	≥200	74	37	1.8 (1.1-2.9)	0.4 (0.2-1.2)	0.100
	<200	102	91	1	1	
TG	≥150	87	34	2.7 (1.6-4.4)	1.2 (0.5-3.1)	0.652
	<150	89	94	1	1	

*significantly associated at p<0.05, 1.0=reference

All lipid profile parameters and fasting blood glucose level were measured in a unit of mg/dl. LDL, Low-Density Lipoprotein; HDL, High-Density Lipoprotein; TC, Total Cholesterol; TG, Triglycerides; FBS, Fasting Blood Sugar; WHR, weight for hip ratio; BMI, Body mass index.

The bold value indicates the p- value for the significant variables.

## Discussion

Diabetes mellitus (DM) is a prevalent noncommunicable disease characterized by significant chronic complications that contribute substantially to the healthcare burden. Contemporary clinical guidelines recommend the use of statins as a preventive measure to mitigate the risk of further diabetes-related complications (8). This study found that only 189 [(53.8%, 95% CI:48.6%, 59.0%)] of eligible individuals with type 2 diabetes mellitus attending the ARTH diabetic clinic, were prescribed statins for the primary prevention of cardiovascular disease. This result is comparable to findings from previous studies conducted in Ethiopia (26), India (27), and Thailand (28) which reported statin prescription rates of 55.7%, 55.2%and 58%, respectively. However, the observed statin utilization rate was higher than those reported in previous studies conducted in Jimma Ethiopia (29.10%) (17). This discrepancy could be due to differences in several risk factors (lipid profile status) and sample size differences. The reference study has smaller sample size as compared to the current study. In addition This finding is also higher to the finding from the study conducted in China (16) 33.8%. the plausible justification could be that this study is conducted in a teaching and referral hospital, where healthcare providers are more likely to follow international guidelines and apply evidence-based practices, particularly in high-risk patients with type 2 diabetes mellitus. In contrast, the Chinese study may have been conducted in in the public hospitals, where guideline adherence can be more variable due to resource constraints or differences in clinical training. To the contrary, this finding is lower than the result of a study conducted in Malaysia (29), which reported that 65% of the participants had statin utilization. This discrepancy could be attributed to differences in healthcare systems, prescribing practices, and population characteristics. In Malaysia, the implementation of national treatment protocols and active engagement by primary care providers may contribute to the higher rate of statin use among appropriate patients (30). Moreover, discrepancies in patient adherence to treatment, societal beliefs regarding cardiovascular disease and pharmacological interventions and the accessibility of lipid-lowering therapies may contribute to the observed variation (31, 32).

According to the study, atorvastatin constituted approximately 99% of all statin therapy prescriptions for primary prevention of CVD in T2DM patients who attended the diabetes clinic, which is in consistent with the findings of a study conducted in India (27). However, this finding contradicts the findings of a study conducted in Ethiopia, where simvastatin was the most frequently prescribed drug (37.2%), followed by atorvastatin (32.8%) and rosuvastatin (15.6%) (26). This variation could be attributed to differences in physician prescribing preference, insurance coverage, or local drug availability (33, 34).

This study aimed to identify the significant predictors influencing statin use for the primary prevention of cardiovascular diseases (CVD). Participants with a history of hypertension were significantly more likely to receive statin prescriptions, This finding is the same as those of a previous study conducted in Eastern Ethiopia (35), Germany (36),Tanzania (34), and the USA (37). This positive association could be justified by the fact that hypertension significantly increases cardiovascular risk, making patients more likely to meet guideline-based criteria for statin therapy (38). Moreover, hypertensive patients typically have more frequent healthcare visits, providing more opportunities for preventive interventions. This frequent contact may also encourage physicians to take timely action to manage their condition effectively and reduce the risk of fatal cardiovascular events.

The findings of the present study also revealed that participants with dyslipidemia had greater odds of statin prescription than did those without dyslipidemia. This pattern of association has also been reported in previous studies conducted in Germany (36), India (27), and Botswana (16). This positive association could be justified by both clinical evidence and guideline-driven practice. Dyslipidemia, particularly elevated LDL-C levels, is a key modifiable risk factor for atherosclerosis, the underlying cause of most cardiovascular events (39, 40). Statins are the first-line treatment to lower LDL-C, making their prescription routine in patients with abnormal lipid profiles. Moreover, dyslipidemia plays a central role in cardiovascular risk assessment tools like the ASCVD risk score (41), which help determine the need for preventive treatment. The presence of elevated lipid levels contributes to a higher calculated risk, often making patients eligible for statin therapy based on clinical guidelines. Routine lipid monitoring and frequent risk evaluations in patients with dyslipidemia further contribute to their higher rates of statin use.

Moreover, statin use was significantly associated with uncontrolled blood sugar level. Statin use is greater in patients with poor glycemic control than in those with controlled glycemic levels. This finding is in line with the finding from the study conducted at Jimma (Ethiopia) (42) and Texas USA (43). This relationship can be explained by the fact that uncontrolled blood glucose level contributes to endothelial dysfunction, oxidative stress, and chronic inflammation that collectively hasten the progression of atherosclerotic cardiovascular disease (ASCVD) (44, 45). Consequently, individuals with poorly managed glycemic levels are considered to be at significantly higher cardiovascular risk, which often leads to the early initiation of statin therapy, even in the absence of classical dyslipidemia. Furthermore, inadequate glycemic control is frequently linked to diabetic dyslipidemia, typically marked by elevated triglycerides, reduced HDL-C, and a higher proportion of small, dense LDL particles (46). These lipid abnormalities further exacerbate cardiovascular risk, thereby increasing the likelihood of statin prescription (47).

On the other hand, several studies have reported that patients taking statins may be at increased risk of developing dyslipidemia and insulin resistance (48–50). This observed association could potentially be linked to the effects of statin therapy; however, due to the cross-sectional nature of our study, it is not possible to establish a definitive cause-and-effect relationship. Hence, the observed association between uncontrolled blood glucose and statin use aligns with evidence-based, risk-oriented clinical guidelines designed to minimize long-term cardiovascular complications in patients with diabetes.

## Conclusion

This study underscores a suboptimal yet comparable rate of statin prescription (53.8%) for primary cardiovascular disease prevention among patients with type 2 diabetes mellitus at the ARTH diabetic clinic, aligning with findings from others lower and middle income countries. Statin use was significantly influenced by clinical factors such as hypertension, dyslipidemia, and poor glycemic control, reflecting adherence to risk-based clinical guidelines. Atorvastatin was the predominant statin prescribed, consistent with international prescribing trends but diverging from local variations. Therefore, it is imperative to implement strategies aimed at optimizing the prescription of statins for the primary prevention of CVD, especially among individuals with comorbidities such as hypertension and dyslipidemia, as well as those with poorly controlled glycemic levels.

### Limitations of the study

While this study has several strengths, it is important to acknowledge its limitations. First, the study design was cross-sectional and limited to a single clinic, which restricts the generalizability of the findings to other healthcare institutions in the country. Moreover, this study design does not allow for the determination of a bidirectional relationship between statin use and the risk of developing dyslipidemia. Additionally, due to the unavailability of certain laboratory investigations, some patients were not fully diagnosed. For instance, the absence of a 24-hour urine protein test at the hospital resulted in many potential cases of diabetic nephropathy being undiagnosed.

### Recommendations

Based on the study findings, it is recommended that institutional guidelines and provider awareness initiatives be strengthened to improve adherence to evidence-based lipid management protocols. Targeted strategies should prioritize high-risk subgroups, including patients with hypertension, dyslipidemia, and poor glycemic control, who are significantly more likely to benefit from statin therapy. Clinical training and continuous medical education focusing on cardiovascular risk stratification and statin prescribing patterns should be emphasized to enhance preventive care practices. Additionally, integrating electronic clinical decision support tools may help identify high-risk individuals and ensure the timely initiation of statins, particularly among those with hypertension, dyslipidemia, or uncontrolled blood glucose levels. Moreover, routine audit and feedback systems should be implemented to monitor and improve prescribing behaviors over time. Future researchers should also focus on investigating the association between dyslipidemia and statin utilization patterns using prospective study designs.

## Data Availability

The original contributions presented in the study are included in the article/supplementary material. Further inquiries can be directed to the corresponding author.
